# Ultrasound with microbubbles

**DOI:** 10.1186/1470-7330-15-S1-O19

**Published:** 2015-10-02

**Authors:** Chris Harvey

**Affiliations:** 1 Imperial College Healthcare NHS Trust, Hammersmith Hospital, London W12 0HS, UK

## 

Conventional ultrasound has well known short comings in the detection and characterisation of focal liver lesions. A false negative rate of up to 30% has been quoted in the detection of liver metastases and its accuracy in the characterisation of focal liver lesions is poor compared to computed tomography (CT) and magnetic resonance (MR). Ultrasound microbubble contrast agents have redressed these limitations.

Ultrasound microbubbles have been available for over 20 years and are licensed for clinical use in most parts of the world. The characterization of focal liver lesions is the most important application of contrast-enhanced US (CEUS), with an accuracy rivalling that of CT and MR [1-8].

Microbubbles consist of a low solubility complex gas such as a perfluoro gas surrounded by a phospholipid shell. They are similar in size to red blood cells, in comparison to the molecular sizes of CT and MR contrast agents. They are pure intravascular agents. Following an intravenous injection they last in the circulation for about 5 minutes. By good fortune, microbubbles resonate in an ultrasound field at the frequencies used in everyday diagnostic sonography. During resonance they emit ‘fingerprint like’ harmonic signals (overtones) which can be selectively detected by the microbubble-specific software available on commercial ultrasound systems. Microbubbles are imaged using low acoustic power modes to reduce their destruction and thus allow real-time imaging. They are better tolerated than MR and CT agents with fewer and less severe adverse effects and are not nephrotoxic. The most widely used agent in Europe is Sonovue (Bracco).

The liver demonstrates three phases of enhancement after an i.v. bolus injection: the arterial, portal and late phases. The late phase occurs as the vascular phases subside, when the microbubbles are sequestered in the sinusoids of the liver.

Enhancement is visualized in real time alongside a B-mode greyscale image. The enhancement characteristics of liver lesions at CEUS are similar to those seen on CT and MR imaging. Since CEUS operates in real time, fast changes during the arterial phase are better captured than on CT or MR.

This presentation discusses the enhancement characteristics of malignant and benign liver lesions. As a general rule, however, lesions which do not washout in the late phase (i.e. remain hyperenhancing or isoenhancing to liver parenchyma) tend to be benign (with the exception of simple cysts, haematomas, ablation cavities and abscesses, which do not enhancement in any phase), whereas lesions which do not retain contrast in the late phase (i.e. demonstrate washout) are usually malignant.

Hepatic metastases have variable appearances on B-mode ultrasound. On CEUS, hypervascular metastases typically demonstrate avid enhancement throughout the lesion in the arterial phase whilst hypovascular metastases show rim enhancement in the arterial phase with both types showing washout in the late phases appearing as defects. CEUS significantly increases the conspicuity of metastases compared to B mode, allowing the detection of isoechoic and lesions down to 3mm.

**CEUS and intervention:** The use of CEUS may allow better visualisation of a lesion than on B-mode ultrasound and allow a targeted ultrasound-guided biopsy.

CEUS may also be used during interstitial ablation of a focal malignant liver lesion. The operator is able to perform repeated injections of microbubbles in order to establish whether viable tumour remains, and whether immediate further on-table ablative therapy is required.

Benign liver lesions have characteristic enhancement patterns on CEUS. **Haemangiomas** demonstrate gradual peripheral nodular enhancement with progressive centripetal filling whereas **focal nodular hyperplasia** typically demonstrate avid arterial centrifugal enhancement in a “spoke wheel” pattern, arising from a central feeding vessel and then become isoenhancing to liver by the late phase.

**Focal Fatty Sparing & Focal Fatty Change** demonstrate identical enhancement to the surrounding liver parenchyma in all phases of CEUS. CEUS can demonstrate enhancing septa in abscesses. CEUS may indicate suitable drainage sites.

In summary microbubbles have expanded the role of US in the real-time characterisation of focal liver lesions based on specific enhancement patterns to differentiate benign and malignant lesions. Ultrasound contrast agents have significantly improved the sensitivity and specificity of ultrasound to rival that of CT and MR.
